# ELF3 is an antagonist of oncogenic-signalling-induced expression of EMT-TF ZEB1

**DOI:** 10.1080/15384047.2018.1507256

**Published:** 2018-08-27

**Authors:** D Liu, Y Skomorovska, J Song, E Bowler, R Harris, M Ravasz, S Bai, M Ayati, K Tamai, M Koyuturk, X Yuan, Z Wang, Y Wang, R.M. Ewing

**Affiliations:** a Department of Oncology, Tongji Hospital, Tongji Medical College, Huazhong University of Science and Technology, Wuhan, China; b School of Biological Sciences, Faculty of Natural and Environmental Sciences, University of Southampton, Southampton, UK; c School of Medicine, Case Western Reserve University, Cleveland, Ohio, USA; d School of Biological Sciences, University of Edinburgh, Edinburgh, UK; e Electrical Engineering and Computer Science, Case Western Reserve University, Cleveland, Ohio, USA

**Keywords:** Oncogenic signalling, epithelial-mesenchymal transition, Wnt signalling, RNA-seq, protein networks

## Abstract

**Background**: Epithelial-to-mesenchymal transition (EMT) is a key step in the transformation of epithelial cells into migratory and invasive tumour cells. Intricate positive and negative regulatory processes regulate EMT. Many oncogenic signalling pathways can induce EMT, but the specific mechanisms of how this occurs, and how this process is controlled are not fully understood.

**Methods**: RNA-Seq analysis, computational analysis of protein networks and large-scale cancer genomics datasets were used to identify ELF3 as a negative regulator of the expression of EMT markers. Western blotting coupled to siRNA as well as analysis of tumour/normal colorectal cancer panels was used to investigate the expression and function of ELF3.

**Results**: RNA-Seq analysis of colorectal cancer cells expressing mutant and wild-type β-catenin and analysis of colorectal cancer cells expressing inducible mutant RAS showed that ELF3 expression is reduced in response to oncogenic signalling and antagonizes Wnt and RAS oncogenic signalling pathways. Analysis of gene-expression patterns across The Cancer Genome Atlas (TCGA) and protein localization in colorectal cancer tumour panels showed that ELF3 expression is anti-correlated with β-catenin and markers of EMT and correlates with better clinical prognosis.

**Conclusions**: ELF3 is a negative regulator of the EMT transcription factor (EMT-TF) ZEB1 through its function as an antagonist of oncogenic signalling.

## Introduction

Epithelial-to-mesenchymal (EMT) transition is a central step in the acquisition of a migratory and invasive phenotype by cancer cells in epithelial tumours, through loss of cell-cell adhesion and cell polarity.^1^ The altered expression of ZEB, Snail and Twist transcription factor families, known as EMT transcription factors (EMT-TFs), that cause destabilisation of adherens junctions and loss of cell-cell adhesion though repression of E-Cadherin is a prominent feature of cells undergoing EMT.^2^ There is significant evidence of cross-talk and feedback between these EMT inducers and oncogenic signaling pathways that activate the EMT process.^3^ For example, transcriptional activation of ZEB1 is induced through Wnt signaling and promoter binding of β-catenin/TCF4.^4,5^ In addition, EMT inducers can enhance oncogenic signaling through multiple molecular mechanisms; Snail can promote expression of Wnt target genes through interaction with β-catenin,^6^ and loss of ZEB1 mediated repression of E-cadherin enables nuclear localization of oncogenic β-catenin.^7^ The balance between regulation of oncogenic signalling and loss of the epithelial state is exemplified by the competition between E-cadherin and the Wnt signalling pathway for β-catenin. Loss of E-cadherin during EMT can release β-catenin and promote β-catenin/TCF driven transcriptional activity and expression of β-catenin–binding domains of E-cadherins can reduce canonical Wnt signalling activity and relocate β-catenin from the nucleus to the plasma membrane.^8^ Despite the depth of study of E-cadherin and its function during EMT, relatively little is known about how the epithelial state is maintained and of factors that repress EMT in cancer cells by antagonizing oncogenic signalling, although transcription factors such as OVOL1, OVOL2^9,10^ and GRLH2^11,12^ that maintain the epithelial state or induce mesenchymal-to-epithelial transition (MET) in human cancers have been described.

Using RNA-Seq analysis of colorectal cancer cells expressing wild-type or oncogenic β-catenin, we identified genes regulated by oncogenic Wnt signalling in colorectal cancer cells. We uncovered the E74-like ETS transcription factor 3 (ELF3), an epithelial-specific transcription factor as significantly down-regulated in cells with oncogenic β-catenin and then investigated the role of ELF3 as an antagonist of oncogenic Wnt signaling and expression of EMT markers. ELF3 is a member of a large family of ETS domain transcription factors that have varied roles in development and oncogenesis and ELF3 itself has been shown to have both oncogenic and tumour suppressor functions, and multiple molecular mechanisms proposed for these activities.^13^ Silencing of ELF3 promotes EMT and increased mobility and invasion of cancer cells,^14^ and a recent study identified ELF3 as a tumour suppressor, with frequent inactivating mutations associated with Wnt pathway activation in ampullary tumours.^15^ In this study, we show that ELF3 expression is repressed by oncogenic Wnt/ β-catenin and RAS signaling and that ELF3 can antagonize oncogenic-signaling induced expression of EMT markers such as ZEB1 (one of the important EMT-TFs) as well as β-catenin/TCF transcriptional activation. In addition to these mechanistic studies, we show that ELF3 gene-expression across tumour panels is inversely correlated to markers of EMT and oncogenic signaling. Analysis of ELF3 in colorectal adenocarcinoma (CRC) tissue microarray panels indicates that ELF3 expression is significantly decreased in colorectal tumour tissues and that the presence of ELF3 expression correlates positively with better clinical prognosis.

## Materials and methods

### Cell line culture and sample extraction

Colorectal cancer cell lines HCT116, HKe3, HKh2, RAS-inducible HKe3 ER:HRAS V12, HCT116-CTNNB1^−/Δ45^ and HCT116-CTNNB1^WT/-^ were regularly maintained in McCoy-5A media (Life Technologies, 16600–108, Carlsbad, CA) containing 10% fetal bovine serum (Life Technologies, 10438–026, Carlsbad, CA) and 1% streptomycin-penicillin (Life Technologies, 15140–148, Carlsbad, CA) at 37°C in CO2 incubator (5% CO2, 100% H2O). Cells were harvested by scraping the cells off plates and then washed with cold PBS twice for immediate use or storage (−80°C). Harvested cells were lysed (25mM Tris-HCl, pH7.4, 1mM EDTA, 150mM NaCl, 1% NP-40, 50% glycerol, Protease inhibitor cocktail) by homogenization and incubated on ice for 30 min followed by centrifugation at 13,000rpm for 30min. The supernatant (soluble fraction) was kept for further analysis. Proteins were quantified by Bio-Rad protein assay dye (500–0006, Bio-Rad, Hercules, CA) by measuring the absorbance at 595nm. RAS-inducible HKe3 ER:HRAS V12 cells express a regulatable RAS construct made up of mutant HRAS fused to the oestrogen receptor (ER) ligand-binding domain that is conditionally responsive to 4-hydroxytamoxifen (OHT).^16^ Addition of OHT acutely activates the RAS pathway in HKe-3 cells expressing ER:HRAS V12.^5^ The ER system uses a mutated estrogen receptor, which does not bind to estrogen, its physiological ligand, but binds with very high affinity to the synthetic ligand 4-OHT and regulates the activity, not expression level of its fusion partner in a 4-OHT-dependent fashion.

### SDS-PAGE & immunoblotting

Western blot analysis was carried out with lysates from cells with urea buffer (8M urea, 1M thiourea, 0.5% CHAPS, 50mM dithiothreitol and 24mM spermine). Primary antibodies were from Sigma (ELF3, 1:500, HPA003479, rabbit polyclonal), Santa Cruz (ZEB1, 1:500, sc-25388, rabbit polyclonal; E-cadherin, 1:1,000, sc-21791, mouse monoclonal 67A4), Abcam (β-tubulin, 1:5,000, ab6046, rabbit polyclonal; β-actin, 1:2,000, ab8226, mouse monoclonal mAbcam 8226), BD Biosciences (β-catenin, 1:2,000, 610154, mouse monoclonal 14/β--catenin), Cell Signaling Technology (phospho-ERK, 1:1,000, 9101, rabbit polyclonal; phospho-Akt-Ser473, 1:1,000, 9271, rabbit polyclonal). Signals were detected using the Odyssey imaging system (LI-COR), and evaluated by ImageJ 1.42q software (National Institutes of Health).

### RNA-seq analysis

The quantity of total RNA in each sample was collected using Qubit (Invitrogen) and libraries prepared using Illumina TruSeq Total RNA v2 kit with Ribo Zero Gold for rRNA removal. The Ribo-Zero kit was used to remove ribosomal RNA (rRNA) from 1 µg of Total RNA using a hybridization/bead capture procedure that selectively binds rRNA species using biotinylated capture probes. The resulting purified mRNA was used as input for the Illumina TruSeq kit in which libraries are tagged with unique adapter-indexes. Final libraries were validated using the Agilent High Sensitivity DNA kit (Agilent), quantified via Qubit, and diluted and denatured per Illumina’s standard protocol. High-throughput sequencing was carried out using the Illumina HiScan SQ instrument, 100 cycle paired-end run, with one sample loaded per lane, yielding on average > 100 million reads per sample. Reads were mapped to human genome hg19 using TopHat2 version 2.1.0^17^ with default settings and reads summarized by gene feature using htseq-count.^18^ Differential expression analysis was performed and p-values adjusted for fdr were computed with DeSeq.^19^ Data are available from GEO (accession: GSE95670).

### RNA-seq and transcription factor analyses

Gene Ontology term enrichment was computed in Pathway Studio v9 (Ariadne Genomics). The significantly differential sets of mutant and wild-type genes from the RNA-Seq analysis were analysed using Enrichr^20^ and oPOSSUM-3.^21^ RNA-Seq high-scoring sub-networks were identified in combined BioGRID (v3.4.136) PPI / RNA-Seq expression networks and predicted transcription factors identified for each significant network. Significant networks were identified using MoBaS as previously described.^22^ MoBaS was used to identify significant sub-networks in PPI network by scoring the sub-networks using the RNA-Seq quantitative measurements. Randomized networks (permutation tests using edge swaps to maintain degree distribution) were then analysed to assess the statistical significance of the identified sub-networks. Each subnetwork deemed significant by MoBaS comprises a group of functionally coherent proteins that exhibit significant aggregate differential expression at the mRNA level.

### Gene-expression data analysis and tools

Colorectal tumour gene-expression datasets were retrieved directly from TCGA^23^ or GEO Accession: GSE41258.^24^ Gene-expression profiles were normalized (unit vector), correlations were computed (Pearson’s r) and clustered using hierarchical clustering (UPGMA). For analysis of ELF3 and CTNNB1 correlations across the TCGA colorectal tumour gene-expression dataset, all correlations across the 20532 genes were computed and ranked. (The top 25 most-correlated ELF3 or CTNNB1 gene-expression profiles are shown in Figure 2B)

**Figure 1:**
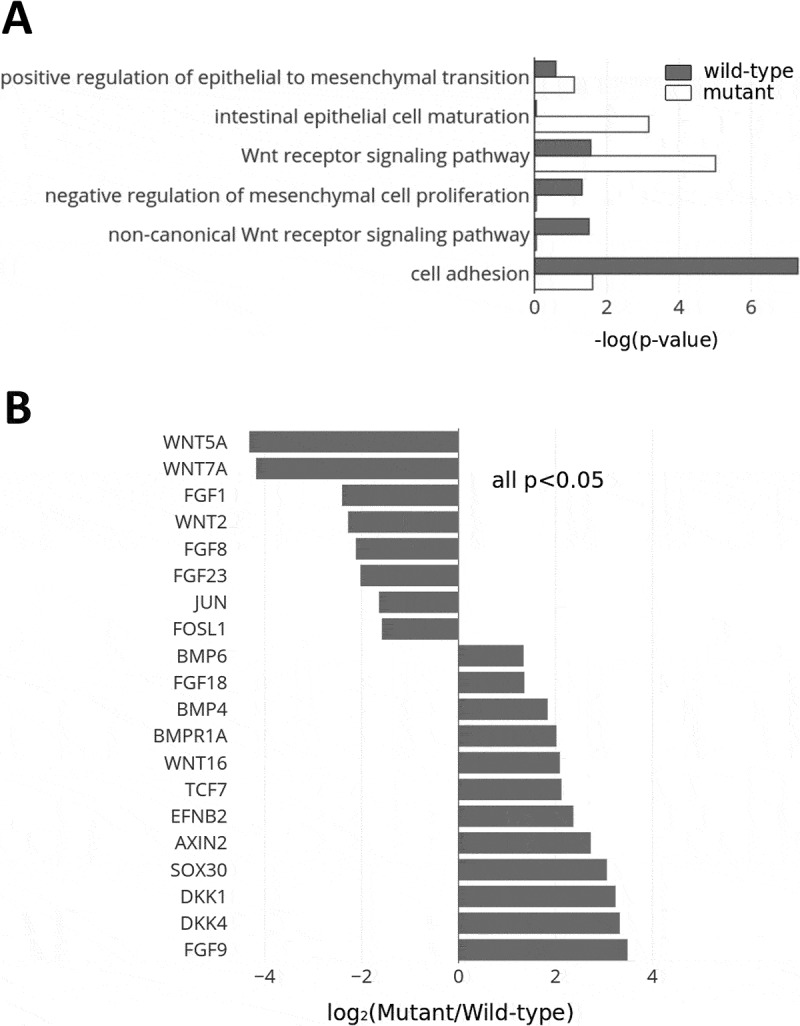
**Comparative transcriptome analysis identifies ELF3 as a candidate regulator of oncogenic signalling-induced EMT (A)** Selected enriched Gene Ontology terms in mutant or wild-type β-catenin cells. Significant Gene Ontology terms (p < 0.05) in either mutant or wild-type cells pertaining to Wnt signalling or EMT are shown **(B, C**) Gene-expression ratios (log2 ratio of mutant/wild-type β-catenin cells from RNA-Seq) for (**B**) known direct β-catenin/TCF transcriptional targets and for (**C**) Epithelial-Mesenchymal-Transition components. Significance (indicated by asterices) is fdr-adjusted Student’s t-test **(D)** Highest scoring sub-network showing connected gene products with significantly differential (p < 0.05) abundance between mutant and wild-type β-catenin cells. Network nodes are coloured according to the mutant/wild-type expression ratio (red = mutant> wild-type), (green = wild-type> mutant). Transcription factors enriched in this network are shown in the Table, and are ranked according to the degree of enrichment. The highest scoring network is significantly enriched for KLF family and ELF3 transcription factors. Asterices indicate those genes predicted to be regulated by ELF3 (from the transcription factor enrichment analysis).

**Figure:**
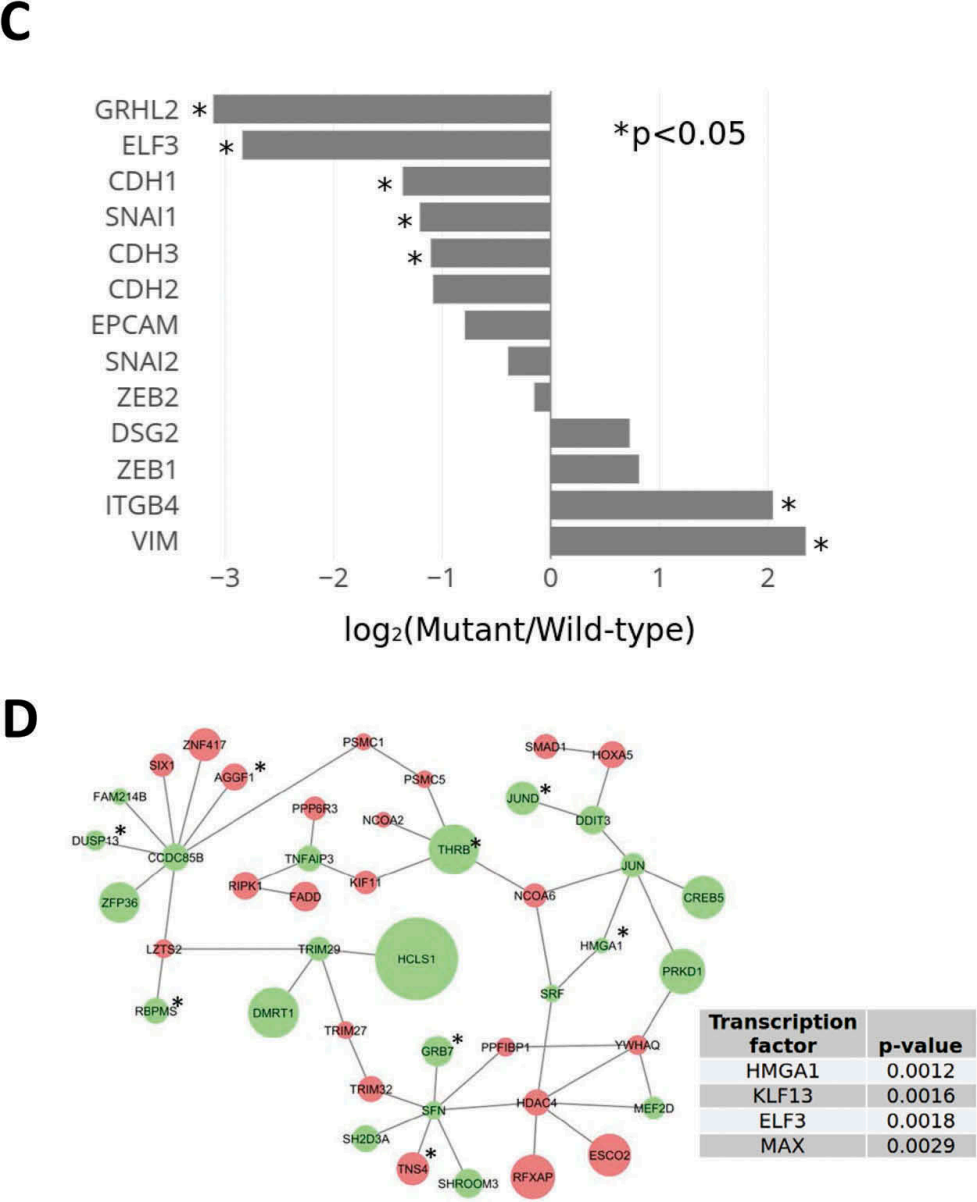
Figure 1 (Continued).

**Figure 2:**
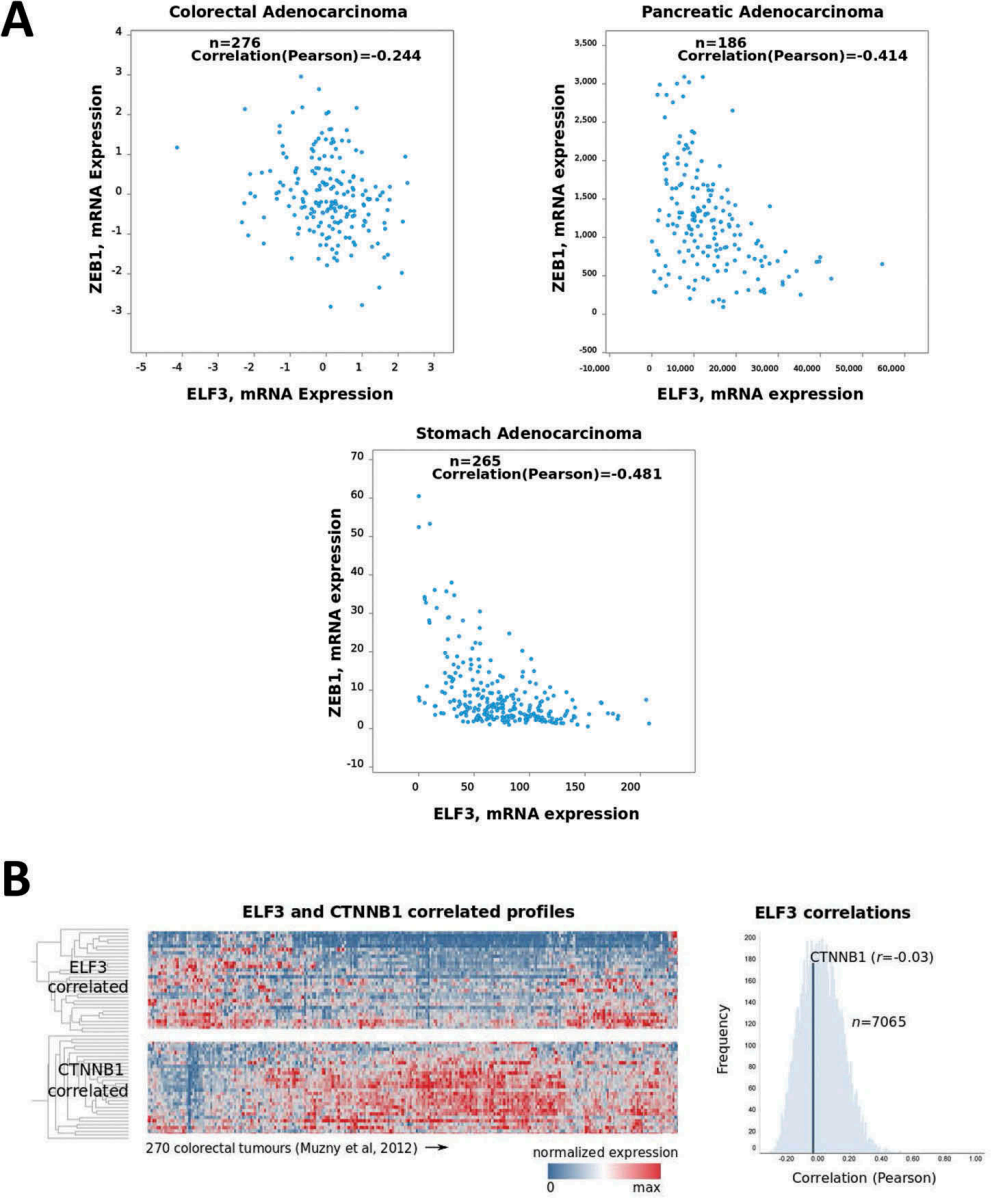
**ELF3 expression and correlation across large-scale tumour datasets (A)** ELF3 and EMT marker ZEB1 show anti-correlation of gene-expression across colorectal, pancreatic and stomach adenocarcinoma TCGA datasets. Colorectal values are microarray z-scores and stomach and pancreatic values are RNA-Seq RSEM values **(B)** Heatmap showing gene-expression values from colorectal cancer TCGA (270 samples) study (Muzny et al, 2012). The 25 most-correlated (Pearson’s r) gene-expression profiles for ELF3 and CTNNB1 are shown as indicated and the histogram indicates the distribution of correlation values across this dataset for ELF3.

### CRC tissue microarray and immunohistochemistry (IHC)

A commercially available tissue microarray (TMA) slide (HColA180Su11, Shanghai Outdo Biotech Co., Ltd., China) with 86 matched pairs of primary CRC samples and adjacent normal tissues was purchased for IHC analysis. All procedures were approved by the Ethical Committee of Tongji Hospital. For IHC, the section was de-waxed, rehydrated and incubated with 3% hydrogen peroxide to block endogenous peroxidase activity (30 min). After microwave antigen retrieval, sections were blocked and then incubated overnight at 4 degree with a primary antibody against ELF3 (1:50, HPA003479, SIGMA), followed by horseradish peroxidase-labelled secondary antibodies for 60 min at 37 degree. After rinsing with PBS three times, staining was visualized using the peroxide substrate solution diaminobenzidine. Counterstained by haematoxylin, the slides were dehydrated in graded alcohol and mounted. Negative controls were prepared in the absence of primary antibody. IHC score was calculated as the multiplication of staining intensity (0 for negative staining, 1 for weak staining, 2 for moderate staining and 3 for strong staining) and the percentage of positive cells (0, < 10% positive cells; 1, 10–30% positive cells; 2, 30–50% positive cells; 3, > 50% positive cells), which range from 0 to 9. Final score < 4 indicated low expression and score ≥ 4 represented high expression. All experiments were performed at least three times. Individual group comparison was performed using Student’s t-test. Kaplan-Meier analysis was used to determine the survival data. Overall survival was defined as the interval between surgery and death, or between surgery and the last observation point. For surviving patients, the data were censored at the last follow-up. *P* value less than 0.05 in all cases was considered statistically significant. All samples were scored blind and the quantitation was limited to the tumour epithelial cells.

### Statistical analysis and reproducibility of experiments

Each experiment was repeated at least twice. Unless otherwise noted, data are presented as mean and s.d., and a two-tailed, unpaired Student’s t-test was used to compare two groups for independent samples. *P* < 0.05 was considered statistically significant.

### Results

To understand how oncogenic signalling drives the transformation of cancer cells, we used a previously described HCT116 colorectal cancer cell model (Chan et al, 2002) expressing either mutant (stabilizing Δ45 mutation) or wild-type β-catenin. Differentially-expressed mRNAs were identified in the mutant (CTNNB1^−/Δ45^) or wild-type (CTNNB1^WT/-^) cells using RNA-Seq which identified 18239 genes with 1085 showing significantly differential expression in mutant cells (p < 0.05; log-fold-change > 2) and 735 showing significantly differential expression in wild-type cells . Gene Ontology (GO) terms significantly (p < 0.05) enriched in mutant and/or wild-type samples were identified. The transcriptome of mutant β-catenin cells is enriched for nuclear and gene-expression associated functions, whereas the wild-type transcriptome is enriched for functions associated with the cell-cell junction role of β-catenin. Gene Ontology terms relevant to Wnt signalling or EMT are shown in Figure 1A, and are substantially different between the mutant and wild-type cells. As expected, highly significant enrichment was observed in the mutant cells for ‘Wnt Receptor Signaling’ (p < 1e-6). We also observed that terms associated with an epithelial phenotype were highly enriched in the wild-type cells (cell-cell adhesion, p < 1e-8; epidermis development p < 1e-6) and that there was some enrichment in mutant cells for genes associated with positive regulation of epithelial-to-mesenchymal transition (p < 0.08).

We next examined the expression of individual genes associated with Wnt signaling and EMT as shown in Figures 1B and 1C. Mutant cells express many markers associated with activation of canonical Wnt signaling, whilst wild-type cells were found to express some non-canonical Wnt signaling components and ligands (e.g. Wnt 5A). Mutant cells express several well-known markers of EMT (VIM, ZEB1) whilst expression of epithelial markers is significantly greater in wild-type cells. We noted that the epithelial transcription factor, E74-Like ETS Transcription Factor 3 (ELF3), was highly differentially expressed between wild-type and mutant cells (~ 8 fold greater in wild-type cells than mutant cells, p < 1e-10). Other genes highly significantly over-expressed in the wild-type cells include GRLH2 (Grainyhead-like transcription factor 2), a known suppressor of oncogenic-induced EMT.^11^ We also analysed an EMT signature previously identified across hundreds of colorectal cancer tumour samples.^25^ Of the signature of 100 genes (46 epithelial, 54 mesenchymal), 29 were significantly differentially expressed in the RNA-Seq data (p < 0.1). The WT-enriched genes associated almost entirely with the epithelial signature (13 out of 16), and the mutant-enriched genes with the mesenchymal signature (8 out of 13), indicating a significant (p = 0.0226, Fisher’s Exact Test) association.

To identify potential transcription factors that drive the observed transcriptomic states, we identified enriched transcription factors in mutant or wild-type cells using the Enrichr tool. Over-represented transcription factors were identified in the significantly differential mutant or wild-type gene-sets (Supplementary data), and the most significant predicted transcription factors identified (Supplementary Figure 1).The wild-type transcriptome was highly enriched in genes regulated by Kruppel-like-factor family (KLF) transcription factors and by ELF3. In addition, ELF3 was a high-frequency occurrence in the set of targets regulated by significant (Enrichr analysis; p < 0.01) transcription factors. To further investigate the significance of these findings, we analysed protein-protein interaction (PPI) networks using a network analysis tool. Significantly differential (p < 0.05) mRNAs were first integrated with the human protein-protein interaction (PPI) network (BioGRID). Significant sub-networks were then identified using modularity-based scoring (MoBaS) to find dense sub-networks of the PPI network with significant aggregate fold change.^22^ Identification of enriched transcription factors in high scoring sub-networks showed that ELF3 and KLF transcription factors were the most significant predicted transcriptional regulators of the top scoring sub-network identified (Figure 1D). In summary, the comparison between cells with mutant or wild-type β-catenin showed a strong epithelial signature in the wild-type cells and induction of EMT in the mutant cells. ELF3 was identified as both significantly greater expressed in the wild-type cells, and one of the most significant predicted transcription factors regulating the observed wild-type transcriptome. We hypothesized that ELF3 may therefore repress oncogenic-signaling induced EMT, and may itself be subject to repression by Wnt/ β-catenin signalling.

We therefore investigated the function of ELF3 and its relationship to oncogenic Wnt signaling in tumour gene-expression datasets as shown in Figure 2. We observed that ELF3 expression is anti-correlated with the expression of the EMT marker, ZEB1, and that this anti-correlation can be observed across multiple tumour types. We observed that ELF3 is also anti-correlated with β-catenin/CTNNB1 (albeit to a less degree). Figure 2B shows heatmap profile plots of the expression of ELF3 and CTNNB1 correlated genes, showing quite distinct and largely mutually exclusive expression profiles. Therefore, the differential expression of Wnt/oncogenic makers versus epithelial markers observed in the mutant/wild-type CTNNB1 cells is also reflected across large-scale tumour gene-expression datasets.

**Figure 3:**
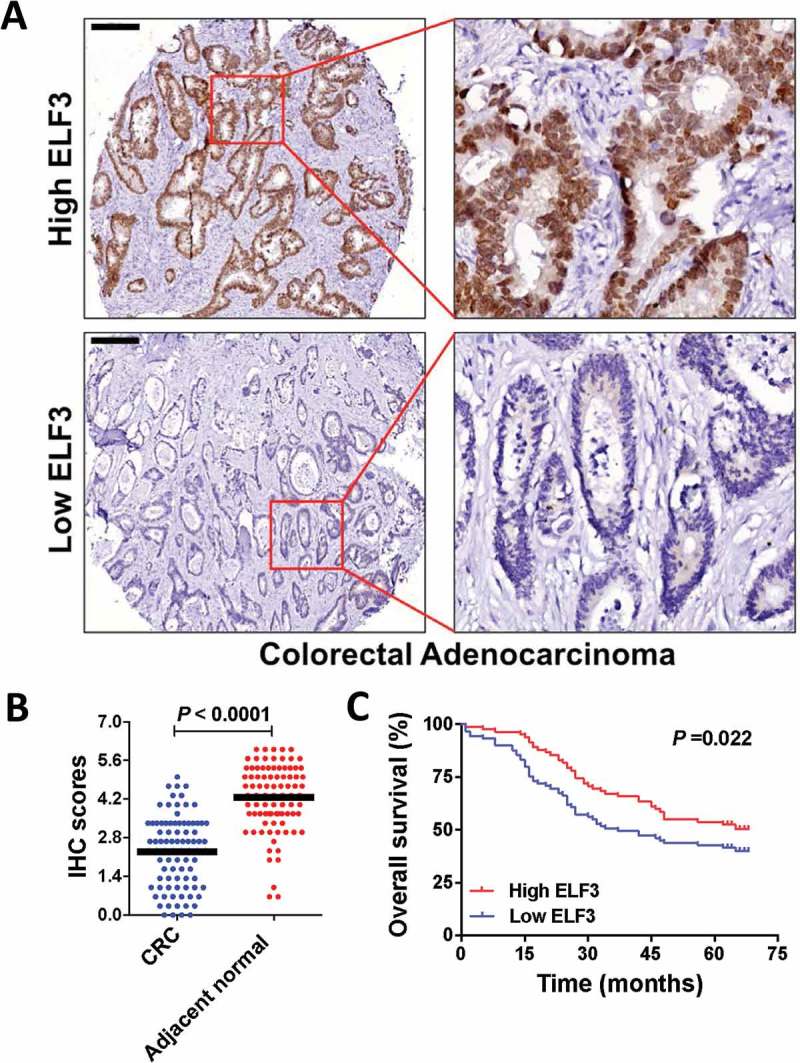
**Reduced ELF3 expression in colorectal adenocarcinoma (CRC) and correlation with poor patient survival (A)** Representative ELF3 staining pattern (High or Low ELF3) in 86 human CRC tissue microarray cores. Scale bar: 200 μm. **(B)** Graph showing IHC scores of ELF3 staining in 86 paired human CRC and adjacent normal tissues (*P* < 0.0001) **(C)** A Kaplan–Meier survival curve shows significant association between low levels of ELF3 and poor survival in CRC patients (*P* = 0.022). The y axis indicates percentage of patients surviving at the indicated time points.

We next investigated the expression of ELF3 protein in colorectal adenocarcinoma (CRC) and adjacent normal tissues (Figure 3 and Supplementary Figure 2). As shown in Supplementary Figure 2, we first observed that ELF3 expression is strongest in epithelial layers of adjacent non-tumour colorectal tissues, and its expression is significantly reduced in CRC (Figure 3A and B). In 86 CRC cores analysed, patients with tumours expressing a high level of ELF3 survived longer when compared with those with tumours expressing a lower level of ELF3 (Figure 3C). These observations agree with the finding that ELF3 may inhibit EMT and tumour progression.

**Figure 4:**
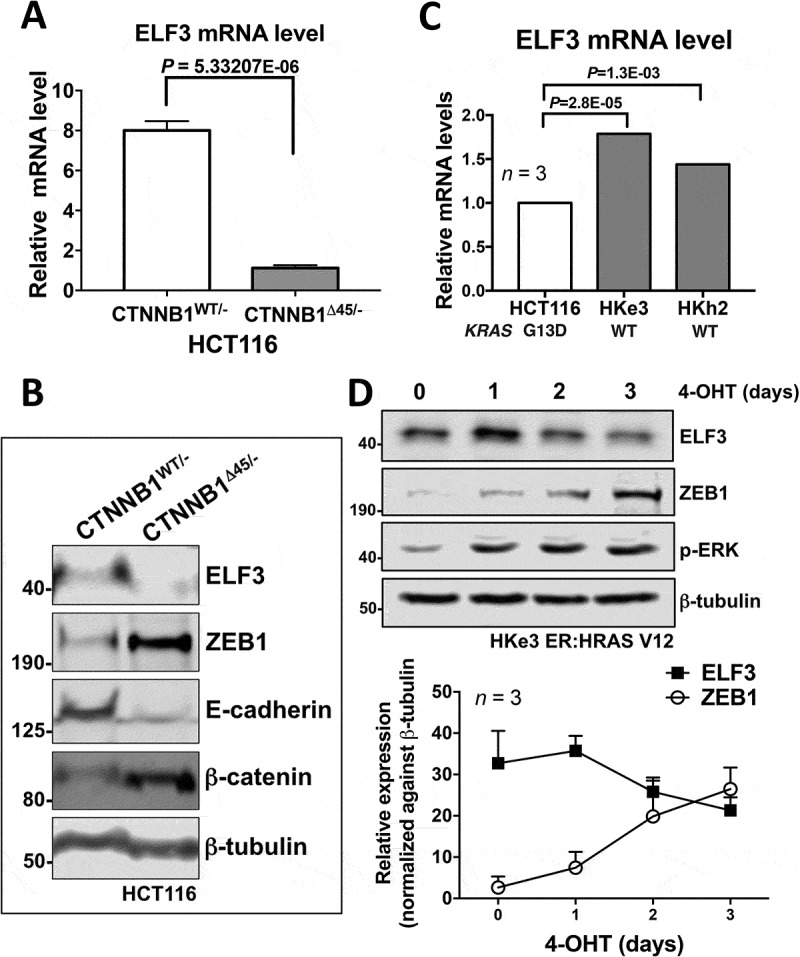
**ELF3 expression is repressed by oncogenic signaling (A)** Relative ELF3 mRNA expression in HCT116-CTNNB1^Δ45/-^ and HCT116-CTNNB1^WT/-^ cells from RNA-Seq analysis (*n* = 3 replicates for each cell-line). **(B)** Western blot analysis of ELF3, β-catenin and EMT markers ZEB1 and E-cadherin in HCT116-CTNNB1^Δ45/-^ and HCT116-CTNNB1^WT/-^ in cell-lines. μ-tubulin was used as a loading control. **(C)** ELF3 mRNA levels in HCT-116 cells compared with HKe-3 and HKh-2 cells, in which mutant KRAS is removed. (*n* = 3 replicates for each cell-line). **(D)** Western blot analysis of ELF3, phosphor-ERK (p-ERK) and an EMT marker ZEB1 in HKe3 ER:HRAS V12 cells with the indicated treatment. β-tubulin was used as a loading control. Graph showing relative expression of ZEB1 or ELF3, normalized to β-tubulin in HKe3 ER:HRAS V12 cells with the indicated treatment (*n* = 3).

To investigate whether ELF3 repression is uniquely associated with oncogenic signalling through the Wnt/β-catenin signaling pathway, we also analysed ELF3 expression in cells with altered CTNNB1 status. Differential expression levels of ELF3 mRNA (Figure 4A) and protein (Figure 4B) were observed in the HCT116-CTNNB1 cell lines, with significantly elevated ELF3 in wild type cells compared to mutant cells, consistent with the RNA-seq results (Figure 1B). In addition, high ZEB1 and low E-cadherin were observed in mutant (CTNNB1-/Δ^45^, compared to wild-type β-catenin (CTNNB1WT/-) HCT116 cells (Figure 4B). To test whether ELF3 repression occurs with other oncogenic signalling pathways, we analysed cells with altered KRAS activity. HCT116 cells carry an endogenous activating KRAS G13D point mutation required for maintaining their oncogenic state. Their isogenic counterparts, HKe−3 and HKh−2, were created by genetic disruption of the activated KRAS allele and are impaired in both anchorage-independent growth and the ability to form tumours in mice.^26^ We found ELF3 is expressed at a higher level in cells with wild type KRAS (HKe-3 and HKh-2) compared to parent HCT116 cells with mutant KRAS (Figure 4C). We then used HKe-3 cells carrying a stable integration of a regulatable RAS construct made up of mutant HRAS fused to the oestrogen receptor (ER) ligand-binding domain that is conditionally responsive to 4-hydroxytamoxifen (OHT).^16^ Addition of OHT acutely activates the RAS pathway in HKe-3 cells expressing ER:HRAS V12.^5,27^ As shown in Figure 4D, a significant reduction in ELF3 protein expression and an increase in ZEB1 level were observed in HKe3 ER:HRAS V12 cells following RAS pathway activation by adding 4-OHT. Therefore, ELF3 repression is associated with the activation of oncogenic signalling via the Wnt/β-catenin or RAS pathways.

**Figure 5:**
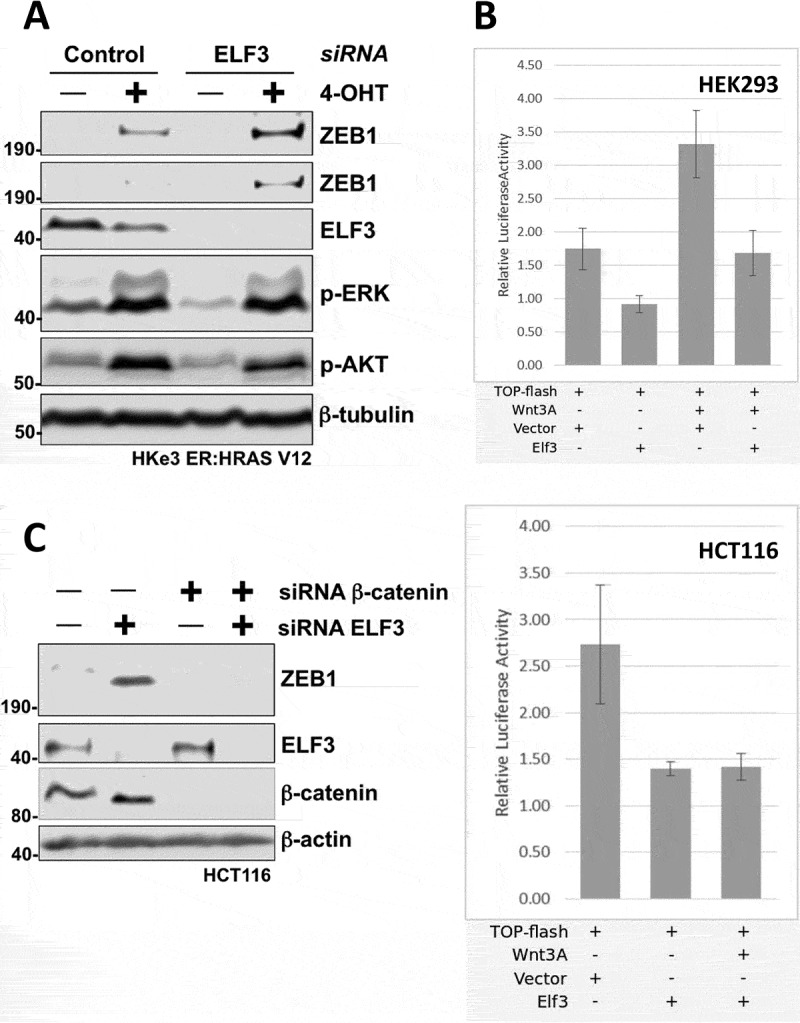
**ELF3 is an antagonist of oncogenic EMT and Wnt signaling (A)** Western blot analysis of ZEB1, ELF3, phospho-ERK (p-ERK) and phospho-AKT (p-AKT) in HKe3 ER:HRAS V12 cells with the indicated treatment. β-tubulin was used as a loading control. **(B)** Expression of human ELF3 inhibits Super8XTOPFlash luciferase reporter activity in HEK293T and in HCT116 cells **(C)** Western blot analysis of ZEB1, ELF3 and β-catenin in HKe3 ER:HRAS V12 cells with the indicated treatment. β-actin was used as a loading control.

Our computational analysis (Figure 2) suggested that ELF3 may repress EMT, in particular ZEB1 expression, in epithelial cells. We showed previously that RAS pathway activation in HKe-3 ER:HRAS V12 induces EMT via up-regulation of ZEB1.^5^ To investigate the role of ELF3 in RAS activation-induced EMT, we knocked down ELF3 in HKe-3 ER:HRAS V12 in the presence or absence of RAS activation by addition of 4-OHT. RAS activation alone had a small but detectable impact on ZEB1 expression. ELF3 knockdown together with oncogenic RAS activation achieved a significant additive effect in inducing EMT, reflected by a large increase in ZEB1 expression (Figure 5A). Given the inverse correlation between ELF3 and β-catenin signatures (Figures 1 and 2), we asked whether ELF3 regulates ZEB1 expression via the Wnt/β-catenin pathway. The effects of ELF3 on Wnt/β-catenin driven transcription were analysed as shown in Figure 5A. ELF3 expression in conjunction with Wnt stimulation (Wnt3A ligand) abrogated the induction of TopFLASH (β-catenin/TCF) responsive reporter expression in HEK293 cells. This was also observed in HCT116 cells, where the addition of exogenous Wnt ligand does not markedly increase the levels of β-catenin nor β-catenin-driven transcriptional activity (Figure 5B).^28^ In HCT116 cells, ZEB1 expression induced by ELF3 RNAi, was abolished on β-catenin knockdown (Figure 5C).

## Discussion

In this study we identified ELF3 as a repressor of the EMT-TF and marker ZEB1 in colorectal cancer cells through its antagonism of Wnt and RAS oncogenic signalling pathways. Transcriptomic analysis of mutant and wild-type β-catenin colorectal cancer cells showed that mutant cells were enriched with mesenchymal markers such as Vimentin (VIM) and that ELF3 was significantly more abundant in wild-type β-catenin cells. We showed that ELF3 exhibits mutually exclusive patterns of expression with CTNNB1/ β-catenin across colorectal tumour panels and that ELF3 is an antagonist of Wnt/β-catenin and RAS signalling-induced EMT.

There has been significant recent interest in the role and function of ELF3 and other members of the ETS-related transcription family in part because of multiple observations of differential gene-expression profiles across cancer tumour datasets. For example, recent analysis of ovarian tumours showed that ELF3 expression was restricted to epithelial cells, and that reduced ELF3 expression across tumour gene-expression panels (TCGA) correlated with poor prognosis.^29^ In the same study, upregulation of ELF3 in ovarian cancer cell-lines caused reduced expression of mesenchymal markers, suppression of proliferation and anchorage-dependent growth. A large-scale genomics screen of ampullary adenocarcinoma patients revealed frequent loss-of-function mutations of ELF3, consistent with its role as a tumour suppressor.^14,15^ In conjunction with these ELF3 inactivating mutations, altered levels of Wnt signalling were observed and it was found that silencing of ELF3 promoted motility and invasion of normal human epithelial cells.

We also investigated the interplay between ELF3, oncogenic signalling via the Wnt and RAS pathways and EMT. Expression of the EMT marker ZEB1 has previously been shown to be transcriptionally regulated by β-catenin/TCF protein complexes in colorectal cancer cells,^30^ and we found elevated ZEB1 in cells with mutant β-catenin. Knock-down of ELF3 in HCT116 colorectal cancer cells substantially increased ZEB1 expression suggesting that ELF3 functions as a repressor of ZEB1 expression. This is abrogated however in cells with concurrent knock-down of both β-catenin and ELF3, suggesting that removal of the ELF3 repression is insufficient, but requires active β-catenin/TCF to induce ZEB1 expression. We also showed that induction of RAS signalling induced ZEB1 expression and decreased ELF3 expression, and that RAS-mediated induction of ZEB1 was enhanced by concurrent knock-down of ELF3, showing that ELF3 represses ZEB1 expression induced by the RAS pathway. In support of its wider role as a transcriptional repressor of oncogenic signalling, ELF3 has been shown to have transcriptional repressor effects in prostate cancer, where it interacts with the androgen receptor, and thereby prevents the recruitment of the androgen receptor to target gene promoters.^31^ In addition, in breast cancer cells, ELF3 has a repressive effect on oestrogen receptor (ERα) transcriptional activity and this results in decreased signalling through this pathway and decreased proliferation.^32^

In contRASt to these studies, ELF3 has also been shown to have an oncogenic role in colorectal cancer, and to be associated with the transactivation of Wnt/ β-catenin signalling.^33^ These authors showed that suppression of ELF3 reduced cell proliferation and Wnt/ β-catenin activation. One feature of ELF3 that may shed light on this apparent paradox may be the differential function of nuclear vs cytosolic ELF3 protein. It was previously shown that ELF3 (ESE-1) is localized in the cytoplasm of human breast cancer cells, and that cytoplasmic ELF3 can induce transformation of human mammary cells whereas nuclear localization of the protein caused apoptosis via a transcription-dependent mechanism.^34^ Furthermore, specific nuclear export sequences are required to maintain the cytoplasmic localization and transformation ability, and the SAR domain alone can initiate transformation.^35^ ELF3 has also been shown to interact with β-catenin and that co-localization of ELF3 and β-catenin occurred in the cytosol and at the plasma membrane.^36^ Clearly, further studies will be required to fully explain the apparently divergent functions of ELF3 in line with the proposal that ELF3 is a moonlighting protein which may have different functions according to the cancer type and cellular localization of the protein.^13^

## Conclusions

In summary, we have showed using analysis of large-scale gene-expression data and from tumour panels that the expression of ELF3 is consistent with its role as an epithelial marker and tumour suppressor in colorectal cancer. We showed that ELF3 is a key transcription factor that differentiates cells with mutant (oncogenic) and wild-type β-catenin, and that ELF3 antagonizes the promotion of ZEB1 expression via the Wnt and RAS oncogenic signalling pathways.

## Abbreviations

ETS: erythroblast transformation specific
EMT: Epithelial-mesenchymal transition
EMT-TF: EMT transcription factor
TCGA: The Cancer Genome Atlas
MET: Mesenchymal-epithelial transition
