# Supplementary figures and images for: ELF3 is an antagonist of oncogenic-signalling-induced expression of EMT-TF ZEB1
Source: Cancer Biol Ther. 2018 Aug 27;20(1):90–100. doi: 10.1080/15384047.2018.1507256 (PMC6292503; doi:10.1080/15384047.2018.1507256)

# Supplementary Figure 1

A

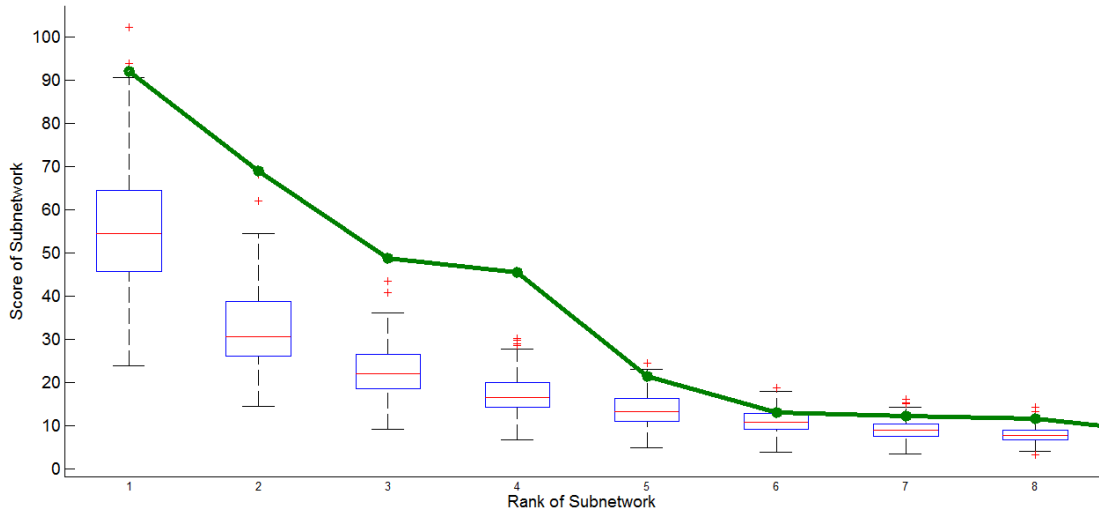

Supplement: Supplemental Material [file kcbt-20-01-1507256-s001.zip › Supplementary information/Figure S1.pdf]

# Supplementary Figure 2

ELF3

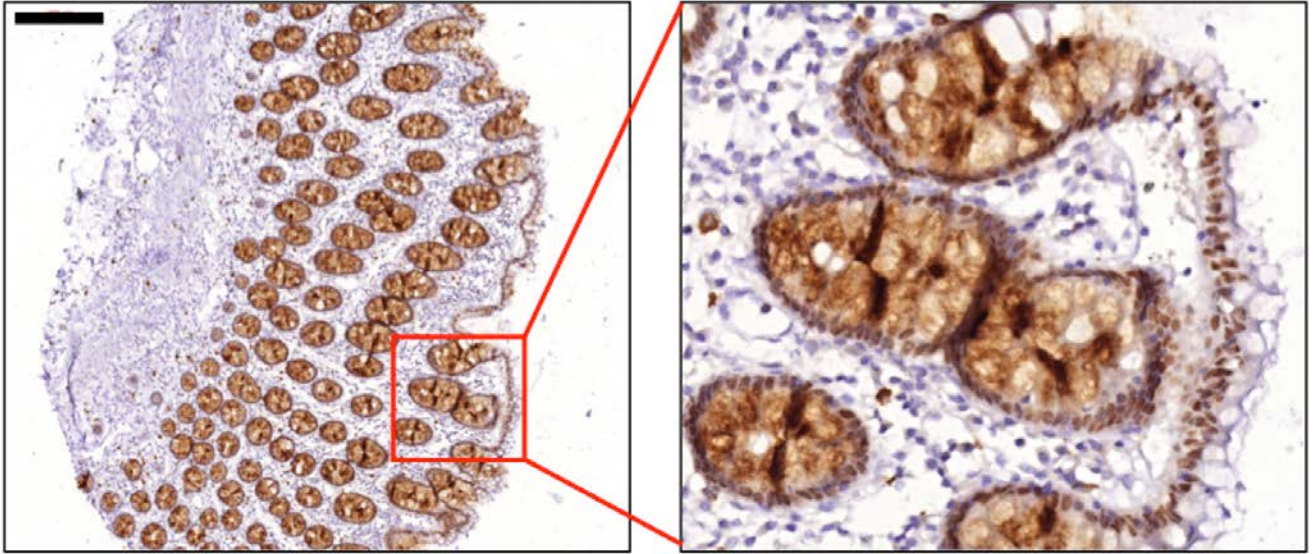

Adjacent normal tissue

Supplement: Supplemental Material [file kcbt-20-01-1507256-s001.zip › Supplementary information/Figure S2.pdf]
